# Early Detection of Liver Fibrosis Using Scatteromics Based on Multimodal QUS Envelope Statistics Imaging

**DOI:** 10.3390/diagnostics16040564

**Published:** 2026-02-13

**Authors:** Ya-Wen Chuang, Duy Chi Le, Chiao-Yin Wang, Dar-In Tai, Zhuhuang Zhou, Po-Hsiang Tsui

**Affiliations:** 1 Department of Biomedical Engineering, Chang Gung University, Taoyuan 333323, Taiwan; ajo729@yahoo.com.tw; 2 Department of Medical Imaging and Radiological Sciences, College of Medicine, Chang Gung University, Taoyuan 333323, Taiwan; duychi346@gmail.com (D.C.L.); everybady123@gmail.com (C.-Y.W.); 3 Department of Gastroenterology and Hepatology, Chang Gung Memorial Hospital at Linkou, Taoyuan 333423, Taiwan; tai48978@cgmh.org.tw; 4 Department of Biomedical Engineering, College of Chemistry and Life Science, Beijing University of Technology, Beijing 100124, China

**Keywords:** ultrasound, scatteromics, liver fibrosis, quantitative ultrasound

## Abstract

**Objectives:** Radiomics has enhanced quantitative ultrasound (QUS) imaging based on envelope statistics for liver fibrosis evaluation. However, early detection of liver fibrosis in patients with hepatic steatosis remains challenging. This study is to develop ultrasound scatteromics prediction models, utilizing simplified feature sets from multimodal QUS envelope statistics imaging, for detecting early-stage liver fibrosis (stage ≥ F1) and significant fibrosis (≥F2) in the presence of hepatic steatosis. **Methods:** The dataset in this prospective study included 252 subjects (*n* = 125 for training and validation; *n* = 127 subjects for independent testing), which underwent blood tests, liver biopsy, and ultrasound radiofrequency data acquisition. In scatteromics analysis, multimodal QUS envelope statistics imaging (Nakagami, homodyned K, and information entropy statistics) was employed. For each imaging, a predefined simplified feature set was calculated, followed by feature selection for machine learning using support vector machine (SVM), random forest (RF), and linear discriminant analysis (LDA). The scatteromics model was obtained using a repeated five-fold stratified cross-validation and then independently tested. The performance was evaluated by the area under the receiver operating characteristic curve (AUROC); scatteromics features were also compared with aspartate aminotransferase (AST) and alanine aminotransferase (ALT). **Results:** Scatteromics features showed no significant correlation with AST and ALT, with correlation coefficients ranging from 0.003 to 0.28. In patients with coexisting hepatic steatosis, scatteromics significantly outperformed QUS envelope statistics imaging in identifying early-stage liver fibrosis, achieving AUROC values of 0.85 to 0.87 for the training and validation datasets, and 0.78 to 0.81 for the testing dataset. In comparison, scatteromics demonstrated modest performance in detecting significant liver fibrosis (≥F2), with AUROC ranging from 0.66 to 0.71 in the training cohort and 0.64 to 0.76 in the testing cohort. **Conclusions:** The proposed scatteromics model streamlines the data analysis workflow of conventional QUS radiomics, enabling early detection of liver fibrosis with reduced dependence on inflammation and hepatic steatosis.

## 1. Introduction

Nonalcoholic fatty liver disease, the most prevalent chronic liver disease globally, includes conditions ranging from hepatic steatosis to hepatocellular carcinoma. Liver fibrosis, often a result of ongoing liver damage, can progress to cirrhosis and cancer if not addressed [1,2]. However, it can be reversible upon removal of the causative agent [3], and some advanced treatments are under development [4]. Early detection of liver fibrosis is crucial for effective management, timely treatment, and reduced mortality from liver diseases.

Noninvasive imaging biomarkers have emerged as a crucial modality for the diagnosis, staging, and longitudinal monitoring of liver fibrosis [5]. Ultrasound serves as the first-line screening tool for the liver, and ultrasound shear-wave elastography (SWE), acclaimed for its ability to evaluate the mechanical properties of tissues, has been recommended as a quantitative evaluation modality for the diagnosis of liver fibrosis [6]. However, the applicability of SWE is significantly curtailed in instances of obesity, ascites, or necroinflammatory activity [7]; in particular, inflammation increases liver stiffness, leading to an overestimation of fibrosis [8].

Compared with SWE, quantitative ultrasound (QUS) imaging based on envelope statistics (i.e., the distribution of echo amplitude), which enables the modeling of ultrasound backscattered statistics for characterizing microstructures in tissues [9], could mitigate the impact of inflammation on the detection of liver fibrosis. The echo amplitude distribution does not exhibit a significant correlation with the grade of necroinflammation activity [10,11]. The Nakagami distribution, homodyned K (HK) distribution, and information entropy are currently well-adopted models for describing envelope statistics of ultrasound backscattering [12]. QUS envelope statistics imaging, utilizing Nakagami [13] and HK parameters [14], has been demonstrated to facilitate the staging of liver fibrosis. Nevertheless, when considering the effect of fat-infiltrated hepatocytes on ultrasound backscattering, the efficacy of QUS envelope statistics imaging in detecting liver fibrosis is contingent upon either excluding subjects with hepatic steatosis or exclusively applying the method to patients with significant hepatic steatosis [13,14].

Note that radiomics extracts extensive features from medical images for diagnostic insights and has been widely adopted for quantitative analysis [15,16]. Integration of ultrasound and QUS imaging with radiomics enhances liver fibrosis evaluation [17,18] and other diseases [19,20,21]. Ultrasomics, a subtype of radiomics using various ultrasound modalities, also improves the discrimination of significant liver fibrosis [22]. However, detecting liver fibrosis in patients with hepatic steatosis using QUS radiomics, including ultrasonics, remains a challenge [18,22]. Another issue with radiomics is that the large number of features derived from advanced mathematical analyses complicates the physical interpretation of imaging. There is growing emphasis on integrating biological and physical insights into radiomics features [23]. Improving the QUS radiomics analysis workflow to enhance both the detection of liver fibrosis in patients with hepatic steatosis and the physical interpretability of the results is an important task that has not yet been fully explored.

We hypothesize that a large number of features may not be required for liver fibrosis detection when multiple QUS models are jointly considered. A recent study introduced the scatteromics technique, which applies machine learning to interpretable, simplified features derived from multimodal QUS envelope statistics imaging for tissue characterization [24]. This study aims to evaluate the performance of scatteromics in detecting early-stage liver fibrosis in patients with hepatic steatosis.

## 2. Materials and Methods

### 2.1. Subject Enrollment

**Figure 1:**
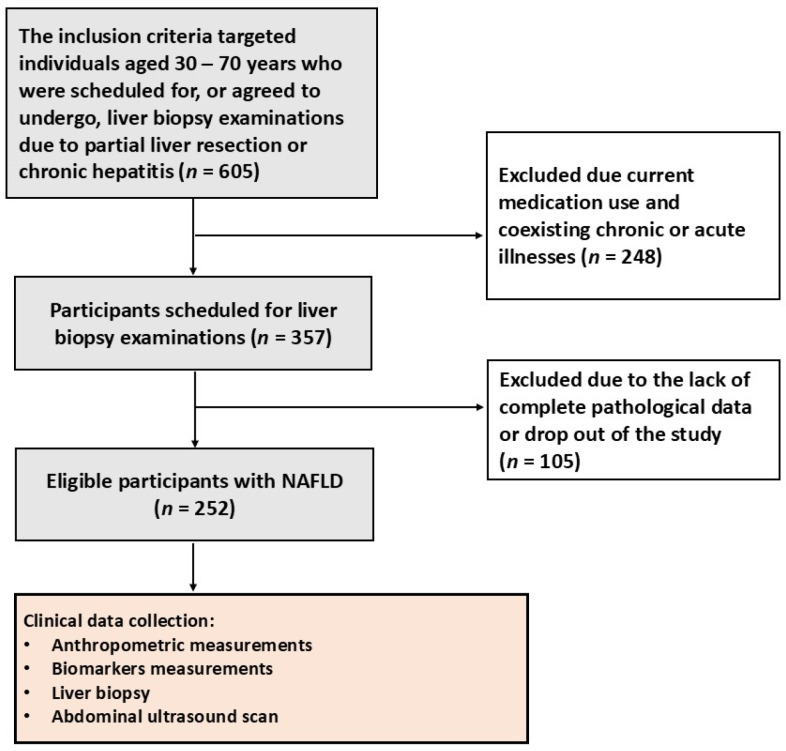
Illustration of the process of study population selection. This study comprised a total of eligible 252 subjects, who underwent anthropometric, biomarkers, liver biopsy, and abdominal ultrasound examinations.

This study received approval from the Institutional Review Board of our hospital. All participants provided signed informed consent forms, and measurement procedures adhered to approved guidelines. The inclusion criteria targeted individuals who were scheduled for, or agreed to undergo, liver biopsy examinations due to partial liver resection or chronic hepatitis. The exclusion criteria encompassed current medication use and coexisting chronic or acute illnesses. This study comprised a total of 252 eligible subjects. The subject enrollment is illustrated in Figure 1.

### 2.2. Subject Measurements

Participants underwent anthropometric measurements and provided venous blood samples after an 8-h fast to measure levels of aspartate aminotransferase (AST) and alanine aminotransferase (ALT). Ultrasound examinations used a clinical system (Model 3000, Terason, Burlington, MA, USA) with a 3.5 MHz convex transducer (Model 5C2A, Terason, Burlington, MA, USA). A radiologist, unaware of the subjects’ clinical data, performed three intercostal liver scans. Each scan captured raw data, including 256 beamformed radiofrequency scan lines during a controlled breath in normal respiration. The sampling rate was set at 12 MHz, with focus and depth at 4 cm and 8 cm, respectively. Pathological examinations followed within a week, using the Metavir scoring system to assess liver fibrosis stages from F0 (no fibrosis) to F4 (cirrhosis) [25]. Hepatic steatosis was graded as normal (less than 5%), mild (5% to 33%), moderate (33% to 66%), or severe (more than 66%) [25]. For the following analyses, early-stage liver fibrosis was defined as ≥F1, and significant liver fibrosis was defined as fibrosis stage ≥F2.

### 2.3. B-Mode and QUS Envelope Statistics Imaging

Ultrasound raw signals were subsequently utilized for B-mode and QUS envelope statistics imaging using the sliding window technique, including four statistical parameters: (i) the Nakagami parameter *m* from the Nakagami distribution, estimated using the moment estimator [13]; (ii) the scatterer clustering parameter *α* from the HK distribution, quantified using the XU estimator [14]; (iii) the coherent-to-diffuse signal ratio *k* derived from the HK distribution, ascertained using the XU estimator [14]; and (iv) the entropy value *H*, determined using the histogram method [12]. The window overlapping ratio was established at 50%, with the side lengths of the window for Nakagami, HK, and entropy parametric imaging designated as three, five, and one times the pulse length of the transducer, respectively [21,26]. Nakagami, HK, and entropy images were superimposed on B-mode images, utilizing pseudocolor coding to concurrently display structural and parametric information. Details regarding the algorithms and physical meanings of Nakagami, HK, and entropy parametric estimations and imaging can be found in previous studies [12,13,14].

### 2.4. Ultrasound Scatteromics Analysis Workflow

Compared with conventional radiomics, scatteromics uses a simplified feature set for feature extraction of multimodal QUS envelope statistics imaging, as opposed to employing filtering or image transformation to generate non-intuitive mathematical features. The detailed algorithm design is described below [24].

Initially, image segmentation was conducted by manually contouring the regions of interest (ROI) related to the liver parenchyma in ultrasound B-scans by an experienced radiologist. The ROI was subsequently placed on each QUS envelope statistics image (i.e., *m*, *α*, *k*, and *H* parametric maps) to calculate 13 first-order statistical measures as a simplified feature set (Table 1), replacing the conventional feature extraction step in radiomics. These measures provide a comprehensive description, enhanced interpretability, and greater flexibility in capturing the statistical nature of QUS envelope statistics imaging. The rationale behind employing multiple envelope statistics models resides in augmenting quantitative information pertaining to scatterer properties, thereby seeking an opportunity to find out potential imaging biomarkers that are associated with liver fibrosis but remain independent of hepatic steatosis.

**Figure 2:**
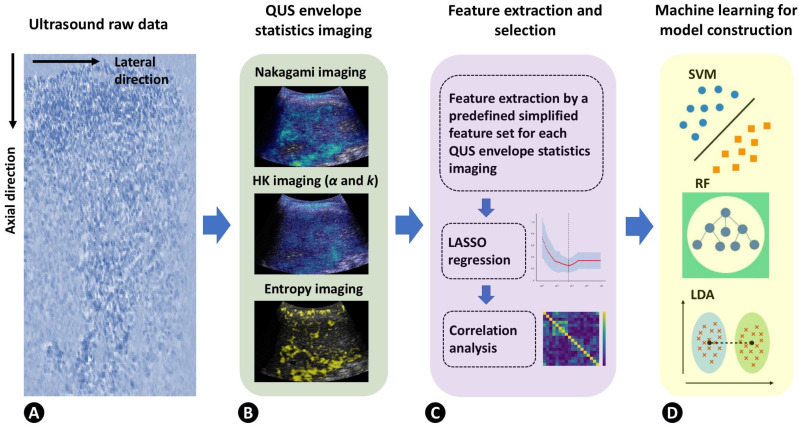
The algorithm of ultrasound scatteromics analysis, including (**A**) using ultrasound raw data as the input; (**B**) QUS envelope statistics imaging is performed using raw data; (**C**) feature extraction by a predefined simplified feature set, and feature selection is performed by LASSO regression and correlation analysis; (**D**) model construction by machine learning.

Subsequently, each feature underwent Z-score normalization, and least absolute shrinkage and selection operator (LASSO) regression was applied. A heatmap was constructed using the correlation coefficient matrix to compare LASSO-retained features against each other. When two features exhibited a Spearman correlation coefficient exceeding 0.8, the feature with the lesser dynamic range was removed. The retained features were then utilized for training and testing in support vector machine (SVM), random forest (RF), and linear discriminant analysis (LDA) classification models. The algorithmic scheme of ultrasound scatteromics is shown in Figure 2.

### 2.5. Training, Validation, and Testing of the Scatteromics Model

The dataset was divided into two subsets: one comprising 125 subjects for training and validation, and another consisting of 127 subjects designated for independent testing. To prevent data leakage, all data splitting was performed at the subject level. Training–validation and testing cohorts were separated by subjects, and cross-validation within the training set was also conducted by subject. Feature selection and model training were performed only within the training subsets, and the independent testing cohort was not involved in model development.

During the training and validation phase, a five-fold stratified cross-validation approach was used for evaluating scatteromics performance. The data was partitioned into five subsets. Four subsets were used for training, and the remaining subset was used for validation (based on an 8:2 ratio). According to the above-mentioned scatteromics analysis workflow, the LASSO regression and correlation analysis were employed to select features based on the training data. Subsequently, the selected features were fed into SVM, RF, and LDA. SVM models were trained using multiple kernels with box constraint values of 0.1–10, and RF models were trained using 100–1000 trees with minimum leaf sizes of 1–20. Hyperparameters were selected based on cross-validation performance within the training data. The model with the lowest cross-validation loss was selected and evaluated on the validation set. LDA models applied discriminant-based feature weighting. The optimal classification threshold was determined using the Youden index. The above process was iteratively conducted to ensure every subset served as a validation dataset at least once, a method that has been shown to produce reliable estimates of model performance [20,21]. To mitigate the impact of data partitioning on the results, we executed 30 separate iterations of five-fold stratified cross-validation using distinct random stratified splits, resulting in a total of 150 training-validation cycles. Notably, feature selection was performed independently within the training subset of each cross-validation fold. For each training-validation cycle, LASSO regression and correlation-based pruning were conducted using only the training data, and the selected features were subsequently applied to the corresponding validation data. The specific features retained in each cycle varied slightly due to the stochastic nature of cross-validation and LASSO regression. To analyze the occurrence of scatteromics features across 150 training-validation cycles, retention probabilities were calculated for all 52 features.

In the testing phase, the scatteromics models that exhibited the highest accuracies during the training-validation cycles were selected to detect liver fibrosis stages in the testing dataset. All tasks were executed using MATLAB software (version R2019a, MathWorks).

### 2.6. Statistical Analysis and Performance Evaluation

Calibration analysis and the Brier score were used to evaluate the reliability of probabilistic outputs generated by the classification models. Calibration curves were constructed using out-of-fold predicted probabilities grouped into ten equally spaced bins. The mean predicted probability within each bin was compared with the observed fraction of positive outcomes, with the 45° reference line indicating perfect calibration. The Brier score was calculated as the mean squared difference between predicted probabilities and observed binary outcomes, with lower values indicating better probabilistic performance.

The parameters *m*, *α*, *k*, and *H* for different liver fibrosis stages were compared using one-way analysis of variance (ANOVA). Correlation coefficients for selected scatteromics features and their relation to AST and ALT levels were visualized with heatmaps, examining their dependence on inflammation. The effectiveness of scatteromics and individual QUS envelope statistics imaging in detecting liver fibrosis stages (≥F1 and ≥F2) was assessed through receiver operating characteristic (ROC) curve. The area under the ROC curve (AUROC) and its corresponding 95% confidence interval (CI) was used for performance evaluations. Statistical analyses were conducted in SigmaPlot (version 12.0, Systat Software, Inc., San Jose, CA, USA). Statistical significance was set at *p* < 0.05.

## 3. Results

**Figure 3:**
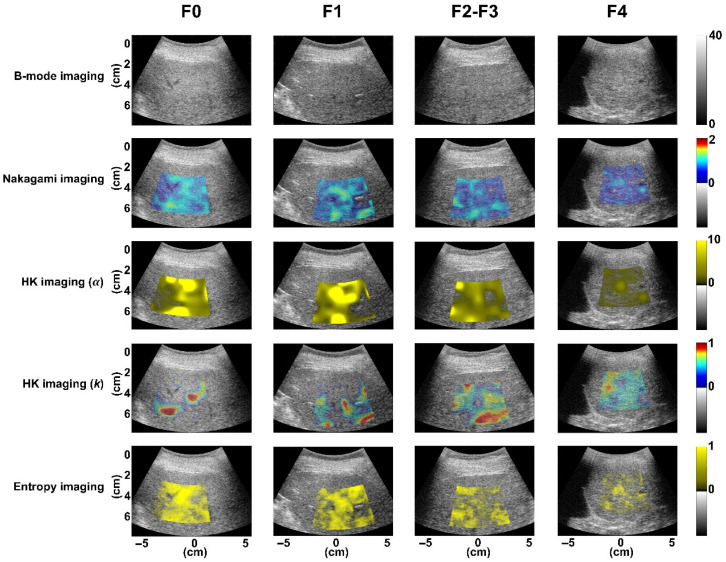
Ultrasound B-mode and QUS envelope statistics imaging (*m*, *α*, *k*, and *H* parametric images) from various stages of liver fibrosis. QUS envelope statistics imaging showed a tendency for decreased brightness as the stage of liver fibrosis advanced.

Demographic data are shown in Table 2. A total of 252 eligible subjects were categorized according to fibrosis stages as follows: F0 (*n* = 148), F1 (*n* = 28), and F4 (*n* = 41). Due to the limited number of samples for stages F2 (*n* = 23) and F3 (*n* = 12), these were combined into a single group, denoted as F2–F3 (*n* = 35). In each fibrosis group, patients also exhibited varying degrees of hepatic steatosis. Figure 3 displays ultrasound B-mode and QUS envelope statistics imaging from different liver fibrosis stages, showing QUS images generally became less bright as fibrosis progressed. As shown in Table 3, there was a decrease in the parameter *m* from 0.82 ± 0.13 to 0.77 ± 0.13, the parameter *α* from 7.43 ± 4.90 to 5.68 ± 3.38, and the parameter *H* from 5.23 ± 0.02 to 5.22 ± 0.03, all correlating with fibrosis advancement (*p* < 0.05). The parameter *k*, however, showed no significant correlation with fibrosis stage (*p* > 0.05).

**Figure 4:**
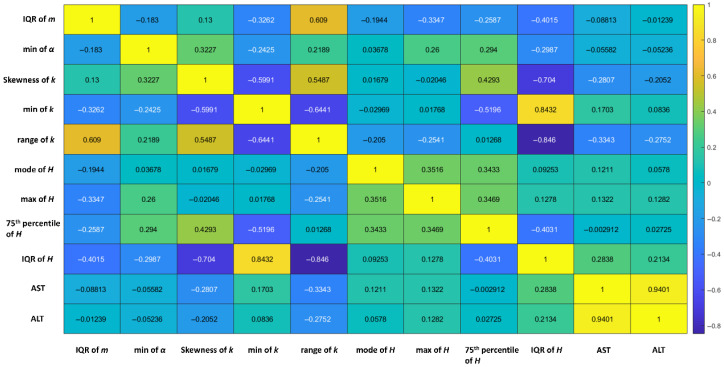
A correlation matrix illustrating the relationships among features retained through LASSO regression, as well as their relationships with AST and ALT. These features include the IQR of the Nakagami parameter, the minimum value of the HK *α* parameter, the skewness of the HK *k* parameter, the minimum value of the HK *k* parameter, the range of the HK *k* parameter, the mode of entropy, the maximum of entropy, the 75th percentile of entropy, and the IQR of entropy. The above features showed low correlations with AST and ALT. The correlation analysis further excluded the minimum of the HK *k* parameter and the range of the HK *k* parameter from feature consideration. Thus, a total of 7 features were selected in this demonstration.

Using the entire dataset, Figure 4 presents a typical correlation matrix of features identified by LASSO regression and their correlations with AST and ALT, with coefficients between 0.003 and 0.28. After performing the correlation analysis to compare features against each other, both the minimum value and the range of the parameter *k* were excluded. In this demonstration, a total of 7 features (the interquartile range, IQR, of the parameter *m*, the minimum value of the parameter *α*, the skewness of the parameter *k*, the mode of *H*, the maximum of *H*, the 75th percentile of *H*, and the IQR of *H*) were selected. Note that these 7 features were not necessarily selected across each training phase but serve to illustrate possible types of features that might be chosen to characterize liver fibrosis.

**Figure 5:**
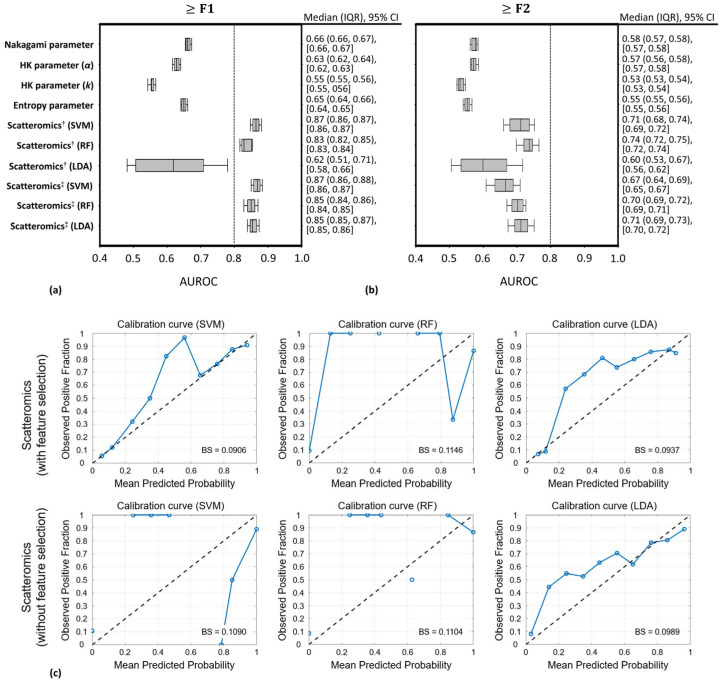
(**a**,**b**) The distribution of AUROCs from 30 iterations of five-fold cross-validation for detections of early-stage liver fibrosis was examined in both QUS envelope statistics imaging and scatteromics (^†^: without feature selection; ^‡^: with feature selection.). (**a**) ≥F1; (**b**) ≥F2. Scatteromics with feature selection significantly outperformed conventional QUS envelope statistics imaging to detect liver fibrosis ≥ F1, achieving AUROCs ranging from 0.85 to 0.87. In detecting liver fibrosis stage ≥ F2, scatteromics showed a reduction in AUROC values, which ranged from 0.60 to 0.74. Meanwhile, QUS envelope statistics parameters resulted in AUROC values that were only between 0.53 and 0.58. (**c**) Calibration performance of the three classification models (SVM, RF, and LDA) using the scatteromics approaches. Calibration curves illustrate the relationship between predicted probabilities and observed outcome frequencies, with the dashed diagonal line indicating perfect calibration. Brier scores (BS) are shown to quantify overall probabilistic accuracy.

Figure 5a,b show the AUROC values, obtained during the training and validation phases, for liver fibrosis detection using QUS envelope statistics imaging and scatteromics. In detecting liver fibrosis at stage ≥ F1, the individual parameters *m*, *α*, *k*, and *H* did not perform well, with median AUROC values ranging from 0.55 to 0.66, although these values varied across fibrosis groups (Table 3). Scatteromics without feature selection achieved AUROC values of 0.87 to 0.83 using SVM and RF methods, but only 0.62 with LDA. Scatteromics with feature selection raised AUROCs ranging from 0.85 to 0.87 across three different machine learning methods. However, in detecting fibrosis ≥ F2, scatteromics dropped to 0.60–0.74 in AUROCs, and QUS envelope statistics parameters showed much lower AUROCs of 0.53–0.58. In Figure 5c, calibration analysis demonstrated that the scatteromics models with feature selection showed relatively good agreement between predicted probabilities and observed outcome frequencies, particularly for the SVM and LDA models, whose calibration curves closely followed the reference diagonal line. Correspondingly, these models achieved lower Brier scores (SVM: 0.0906; LDA: 0.0937) compared with RF (0.1146), indicating better probabilistic accuracy. For scatteromics models without feature selection, calibration performance was slightly reduced, as reflected by increased deviations from the reference line and higher Brier scores (SVM: 0.1090; RF: 0.1104; LDA: 0.0989). Among these models, LDA maintained relatively stable calibration performance compared with SVM and RF. Overall, feature selection improved probabilistic calibration, and SVM and LDA models demonstrated more reliable probability estimation.

**Figure 6:**
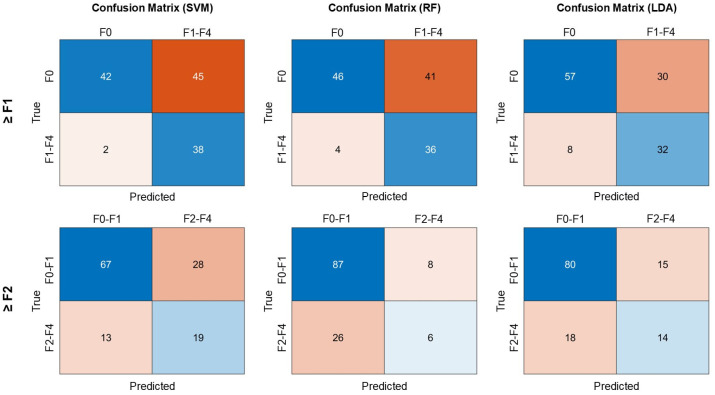
Confusion matrices of the scatteromics-based classification models for detecting early-stage fibrosis (≥F1) and significant fibrosis (≥F2) in the testing cohort. The upper panels present results for ≥F1 detection using SVM, RF, and LDA models, while the lower panels present results for ≥F2 detection. Rows indicate true fibrosis status and columns indicate predicted classification. Numbers within each cell represent the number of subjects in each category.

The results from the independent testing dataset are shown in Table 4 and Figure 6. The AUROC values for detecting liver fibrosis at stage ≥ F1 using scatteromics models based on SVM, RF, and LDA were 0.81, 0.78, and 0.79, respectively. All three models demonstrated high sensitivity, with SVM showing the lowest number of false negatives, whereas LDA showed relatively fewer false-positive classifications. For detecting liver fibrosis at stage ≥ F2, the AUROCs for models based on SVM, RF, and LDA were 0.72, 0.64, and 0.76, respectively. All models demonstrated reduced diagnostic performance.

**Figure 7:**
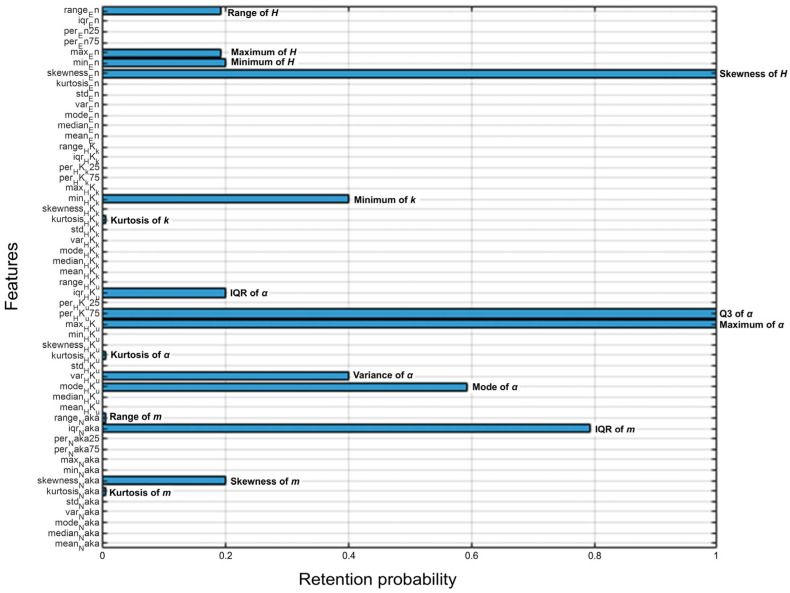
Retention probabilities of 52 first-order statistical features across 150 training-validation cycles. A total of 16 features were retained.

Figure 7 shows the retention probabilities of 52 first-order statistical features across 150 training–validation cycles. A total of 16 features were retained across these cycles. Skewness of *H* and Q3 and maximum of α showed retention probabilities of 100%, indicating their consistent importance in the model. In addition, several features demonstrated retention probabilities greater than 50%, including IQR of m and mode of α.

## 4. Discussion

Conventional QUS radiomics faces challenges in characterizing liver fibrosis due to the use of a single model and the influence of hepatic steatosis. In normal liver tissue, backscattered envelope statistics typically conform to a Rayleigh distribution [27]. However, the liver’s complex vascular structure often leads to a deviation from the Rayleigh distribution, resulting in a pre-Rayleigh distribution in practical observations [14,28]. Liver fibrosis increases the variance in the scattering cross-sections of scatterers, shifting the echo amplitude distribution towards a more pronounced pre-Rayleigh pattern [29], which corresponds to decreases in the *m*, *α*, and *H* parameters. On the other hand, hepatic steatosis, characterized by fat-infiltrated hepatocytes, increases the number of scatterers, promoting constructive wave interference. This shifts the backscattered statistics from a pre-Rayleigh distribution closer to a Rayleigh distribution [12,26,30], leading to increases in the *m*, *α*, and *H* parameters. Because signals backscattered from steatosis are empirically stronger than those from fibrotic regions [14], in cases where steatosis and fibrosis coexist, fat-infiltrated hepatocytes become the dominant scatterers, affecting the performance of QUS envelope statistics imaging in liver fibrosis detection.

Scatteromics may help address the limitations of conventional QUS techniques. The current findings indicate that scatteromics features are less influenced by liver inflammation, and the derived prediction models effectively detect early-stage liver fibrosis in patients with coexisting hepatic steatosis. In the training and validation datasets, AUROC values ranged from 0.85 to 0.87. The SVM model achieved a sensitivity of 88.93%, with RF and LDA also performing well. In terms of specificity, SVM reached 77.46%, slightly higher than RF and LDA. Overall accuracy ranged from 80.08% to 83.31%. In the testing dataset, AUROC values slightly decreased to a range of 0.78 to 0.81. The SVM model achieved a high sensitivity of 95%, but its specificity was lower at 48.28%. Compared to SVM and RF, LDA had a higher specificity at 65.52%. Accuracy in the testing dataset was lower than in the training and validation datasets, with LDA achieving the highest accuracy at 70.07%. The testing dataset showed a decrease in AUROC, accuracy, and specificity, indicating slightly reduced model performance on unseen data. This drop in performance may be due to model overfitting or greater heterogeneity in the testing data. However, the high AUROC values in the testing dataset still demonstrated that scatteromics has strong potential in real-world applications, particularly for identifying early-stage fibrosis with high sensitivity, making it useful for early screening. On the other hand, it should be noted that BMI was higher in the F0 group and may potentially influence ultrasound signal propagation and attenuation. However, scatteromics features are derived from QUS envelope statistics imaging, in which pixel values represent statistical properties of backscattered echoes rather than backscatter amplitude alone. Therefore, scatteromics primarily reflects tissue microstructural scattering characteristics, and the influence of BMI on these features may be limited.

The demonstration using the entire dataset identified seven features that may help interpret the underlying physics of scatteromics in liver fibrosis. The IQR may effectively highlight changes in scatterer concentration, as depicted by the Nakagami parameter. The minimum value of the HK *α* parameter may indicate the highest likelihood of liver fibrosis. Additionally, specific features such as the skewness of the HK *k* parameter and certain entropy metrics (mode, maximum, 75th percentile, and IQR) were also selected by scatteromics analysis. The skewness of the HK *k* parameter measures the asymmetry in the distribution of the coherent-to-diffuse scattering power ratio, reflecting the formation of fibrotic structures within the liver. Similarly, entropy metrics may help characterize the randomness or complexity of the signal, which is associated with tissue heterogeneity arising from liver fibrosis.

Scatteromics may complement ultrasound elastography in liver disease management. Significant liver fibrosis and cirrhosis are well-established predictors of increased liver disease-related mortality [31]. Numerous studies have demonstrated that ultrasound elastography has high diagnostic accuracy for significant liver fibrosis; however, its efficacy in detecting early-stage fibrosis remains inconsistent and lacks consensus [32,33]. The need for early intervention to prevent fibrosis underscores the importance of more advanced diagnostic tools [34]. The current results suggest that scatteromics is effective in identifying early-stage liver fibrosis, indicating that scatteromics could work in conjunction with elastography to improve diagnostic accuracy across all stages of liver fibrosis. Moreover, in the current risk stratification and management of patients with nonalcoholic fatty liver disease, ultrasound imaging is commonly used as a first-line modality to assess hepatic steatosis. Because the scatteromics framework is compatible with diagnostic ultrasound systems, integration of this framework may enable preliminary assessment of liver fibrosis risk during routine steatosis screening, thereby enhancing existing risk stratification strategies.

In addition, scatteromics offers clinical benefits by simplifying the complexity of feature extraction compared to conventional radiomics, streamlining the analysis of clinical liver data. By utilizing a simplified feature set combined with multimodal QUS envelope statistics models, the results indicate that, despite varying degrees of hepatic steatosis in patients, the early detection of liver fibrosis remains effective. This suggests that in future QUS clinical applications, it may not be necessary to pre-classify patients based on the severity of hepatic steatosis or to establish distinct diagnostic criteria or thresholds for liver fibrosis across different levels of fatty liver.

This study has several limitations. First, the single-center design and use of a single ultrasound platform may limit generalizability. The imbalanced fibrosis stage distribution and relatively small sample sizes in certain groups may have influenced model performance. The relatively low specificity observed in the testing dataset indicates a higher rate of false-positive results, which could limit the clinical utility of scatteromics as a standalone diagnostic tool. Future studies will require a larger cohort to improve performance and reduce the occurrence of false positives. Second, improving performance will also require advancements in methodologies. Currently, only the Nakagami, HK, and entropy approaches were explored in the scatteromics analysis. Incorporating additional distributions or alternative models may further enhance the diagnostic accuracy and robustness of the scatteromics framework. Third, the current scatteromics algorithm demonstrated advantages in the detection of early-stage liver fibrosis, whereas ultrasound elastography has been widely recognized as an effective tool for evaluating significant fibrosis. Therefore, scatteromics and elastography (e.g., SWE) should be considered complementary rather than competing approaches. Future studies integrating scatteromics with elastography may provide a more comprehensive and clinically practical diagnostic framework covering both early and significant stages of liver fibrosis. Finally, due to the current lack of external validation, clinical implementation of the proposed scatteromics framework should be approached cautiously, and further validation in independent and multicenter cohorts is required to confirm its generalizability.

## 5. Conclusions

In summary, the proposed scatteromics model streamlines the data analysis workflow of conventional QUS radiomics, enabling early detection of liver fibrosis with reduced sensitivity to inflammation and hepatic steatosis. Scatteromics also improves data interpretability by establishing a link between prediction models and the physical insights derived from imaging.

## Figures and Tables

**Table 1:** In the scatteromics analysis, a simplified feature set, comprising 13 first-order statistical measures, is used for feature extraction of multimodal QUS envelope statistics imaging. These measures include mean, median, mode, variance, standard deviation, kurtosis, skewness, minimum, maximum, 75th percentile (Q3), 25th percentile (Q1), IQR, and range. In mathematical formulas, the symbols *x*_1_, *x*_2_, *x*_3_, …*x*_n_ are the individual numbers in the dataset, *μ* represents the mean, and *σ* represents the standard deviation.

Features	Definition	Formula
Mean	The average of a set of values that is calculated by summing all values and dividing by the total number of values.	$\mu =\frac{1}{n}\sum_{i=1}^{n}x_{i}$
Median	The middle value in a dataset when it is ordered from lowest to highest; it is not affected by extreme outliers.	
Mode	The value that appears most frequently in a dataset.	
Variance	Variance measures the spread or dispersion of data points from the mean and is calculated by averaging the squared differences between each data point and the mean.	$Variance=\frac{1}{n}\sum_{i=1}^{n}{\left(x_{i}-\mu \right)}^{2}$
Standard deviation	A measure of the average distance between each data point and the mean; it is the square root of the variance.	$\sigma =\sqrt{variance}$
Kurtosis	A measure for the tailedness of a probability distribution, indicating whether a dataset has heavier or lighter tails compared to a normal distribution.	$Kurtosis=\frac{1}{n}\sum_{i=1}^{n}{\left(\frac{x_{i}-\mu }{\sigma }\right)}^{4}-3$
Skewness	A measure for the asymmetry of the probability distribution of a dataset; positive skewness indicates a longer right tail, while negative skewness indicates a longer left tail.	$Skewness=\frac{1}{n}\sum_{i=1}^{n}{\left(\frac{x_{i}-\mu }{\sigma }\right)}^{3}$
Minimum	The smallest value in a dataset.	
Maximum	The largest value in a dataset.	
75th percentile (Q3)	The value below which 75% of the data falls.	
25th percentile (Q1)	The value below which 25% of the data falls.	
Interquartile range (IQR)	The range between the 75th percentile and the 25th percentile and represents the middle 50% of the data.	IQR=Q3−Q1
Range	The difference between the maximum and minimum values in a dataset, indicating the full spread of the data.	Range=Maximum−Minimum

**Table 2:** Demographic data of the participants enrolled in this study.

Fibrosis Stage	F0	F1	F2–F3	F4	F1–F4
Number of patients	148	28	35	41	104
Age (year)					
Mean ± standard deviation	57.78 ± 1.19	48.14 ± 12.32 *	53.94 ± 10.51	54.07 ± 11.39	52.43 ± 11.56 *
Median (IQR)	61.00 (50.00–67.00)	49.00 (36.00–56.75)	52.00 (47.00–60.00)	55.00 (46.00–65.00)	52.00 (45.00–60.00)
BMI ($kg/m^{2}$)					
Mean ± standard deviation	33.42 ± 21.47	24.11 ± 4.09	23.84 ± 3.13 *	27.07 ± 4.97 **	25.19 ± 4.42
Median (IQR)	25.89 (23.48–30.05)	22.92 (21.23–26.96)	23.73 (21.44–25.59)	26.60 (23.30–29.70)	24.09 (21.97–27.87)
ALT (U/L)					
Mean ± standard deviation	34.13 ± 44.65	198.25 ± 446.19 *	219.59 ± 221.30 *	74.78 ± 44.48 *	151.83 ± 278.86 *
Median (IQR)	20.00 (14.00–35.00)	86.00 (38.50–110.50)	89.00 (48.00–400.00)	64.00 (47.00–102.00)	80.50 (46.25–118.00)
AST (U/L)					
Mean ± standard deviation	29.64 ± 22.79	94.09 ± 165.66 *	109.46 ± 119.58 *	59.81 ± 38.65 *	85.75 ± 113.89 *
Median (IQR)	23.00 (17.00–33.00)	48.50 (35.25–79.75)	56.00 (33.00–130.00)	51.00 (30.50–72.00)	51.50 (34.00–86.00)
Hepatic steatosis grade					
Normal (<5%)	32 (32%)	12 (43%)	18 (52%)	15 (37%)	45 (43%)
Hepatic steatosis	69 (68%)	16 (57%)	17 (48%)	26 (63%)	59 (57%)
Mild (5–33%)	18 (18%)	14 (50%)	12 (34%)	10 (24%)	36 (35%)
Moderate (33–66%)	22 (22%)	2 (7%)	1 (3%)	6 (15%)	9 (9%)
Severe (>66%)	29 (28%)	0 (0%)	4 (11%)	10 (24%)	14 (13%)

IQR: interquartile range; BMI: body mass index; ALT: alanine aminotransferase; AST: aspartate aminotransferase. *: *p* < 0.05 compared with F0; **: *p* < 0.05 compared with F2–F3.

**Table 3:** QUS envelope statistics parameters obtained from the *m*, *α*, *k*, and *H* parametric images at different stages of liver fibrosis (the training dataset). Significant difference was found between different stages of liver fibrosis for the *m*, *α*, and *H* parameters. The Nakagami parameter and the HK *α* parameter exhibited variations across liver fibrosis stages, offering a broader dynamic range for detection compared to the HK *k* and entropy values.

Fibrosis Stage	F0	F1	F2–F3	F4	*p* Value (ANOVA)
Nakagami parameter					
Mean ± standard deviation	0.82 ± 0.13	0.73 ± 0.06	0.73 ± 0.08	0.77 ± 0.13	p<0.05
Median (IQR)	0.84 (0.72–0.94)	0.73 (0.69–0.77)	0.73 (0.66–0.80)	0.80 (0.72–0.84)	
HK parameter (α)					
Mean ± standard deviation	7.43 ± 4.90	4.18 ± 1.43	4.05 ± 1.87	5.68 ± 3.38	p<0.05
Median (IQR)	6.32 (3.12–11.72)	3.94 (3.09–5.04)	3.79 (2.44–5.59)	5.57 (3.16–6.96)	
HK parameter (*k*)					
Mean ± standard deviation	0.44 ± 0.09	0.41 ± 0.06	0.40 ± 0.07	0.43 ± 0.05	p>0.05
Median (IQR)	0.43 (0.37–0.51)	0.42 (0.38–0.45)	0.39 (0.35–0.46)	0.42 (0.40–0.47)	
Entropy parameter					
Mean ± standard deviation	5.23 ± 0.02	5.21 ± 0.02	5.21 ± 0.02	5.22 ± 0.03	p<0.05
Median (IQR)	5.23 (5.22–5.24)	5.21 (5.20–5.23)	5.22 (5.19–5.23)	5.23 (5.21–5.24)	

**Table 4:** Performance evaluation of ultrasound scatteromics with feature selection in detecting liver fibrosis. Data from the training and validation phases are expressed as mean ± standard deviation. For the testing results, data is reported as a single testing outcome.

	≥F1						≥F2					
Model	Training/Validation			Testing			Training/Validation			Testing		
	SVM	RF	LDA	SVM	RF	LDA	SVM	RF	LDA	SVM	RF	LDA
AUROC	0.87	0.85 *	0.86 *	0.81	0.78	0.79	0.66	0.70 *	0.71 *	0.72	0.64	0.76
Accuracy (%)	83.31	80.08 *	81.28 *^,^**	63.00	64.57	70.07	62.72	63.04	63.41	67.72	73.23	74.02
Sensitivity (%)	88.93	86.09 *	85.98 *	95.00	90.00	80.00	47.38	38.10 *	71.60 *^,^**	59.38	18.75	43.75
Specificity (%)	77.46 **	73.85	76.44 **	48.28	52.87	65.52	71.14	76.60 *	58.97 *^,^**	70.53	91.58	84.21
F1-score	0.85	0.82 *	0.82 *	0.62	0.62	0.63	0.45	0.40	0.57 *^,^**	0.48	0.26	0.46
LR+	5.03 **	4.02	4.95 **	1.84	1.91	2.32	1.77	1.85	1.84	2.01	2.23	2.77
LR–	0.14	0.19 *	0.18 *	0.10	0.19	0.31	0.73	0.81 *	0.46 *^,^**	0.58	0.89	0.68
PPV (%)	81.35 **	78.34	80.32 **	45.78	46.75	51.61	45.47	46.32	48.65	40.43	42.86	48.28
NPV (%)	87.42	84.25 *	84.85 *	95.45	92.00	87.69	72.38	69.91 *	81.14 *^,^**	83.75	76.99	81.63
Balanced accuracy (%)				71.63	71.43	72.75				64.95	55.16	63.98

Data are expressed as mean ± standard deviation. SVM: support vector machine; RF: random forest; LDA: linear discriminant analysis; AUROC: area under the receiver operating characteristic curve; LR+: positive diagnostic likelihood ratio; LR–: negative diagnostic likelihood ratio; PPV: positive predictive value; NPV: negative predictive value. *: *p* < 0.05 compared with SVM; **: *p* < 0.05 compared with RF.

## Data Availability

The data presented in this study are available on request from the corresponding author.
